# Studies on polynuclear furoquinones. Part 1: Synthesis of tri- and tetra-cyclic furoquinones simulating BCD/ABCD ring system of furoquinone diterpenoids

**DOI:** 10.3762/bjoc.5.47

**Published:** 2009-09-29

**Authors:** Faruk Hasan Shaik, Gandhi Kumar Kar

**Affiliations:** 1 Department of Chemistry, Presidency College, 86/1 College Street, Kolkata-700073, India, Tel: +9133 2241-3893

**Keywords:** Fremy’s salt oxidation, furonaphthoquinone, furophenanthraquinone, Suzuki reaction

## Abstract

Synthesis of phenanthro[1,2-*b*]furan-10,11-dione, the core nucleus present in Tanshinone-I is described in 8–10 steps starting from 2-bromo-3,4-dihydro-1-naphthaldehyde. The bromoaldehyde was converted to methyl 2-(2-bromo-1-naphthyl)acetate or 2-(2-bromo-1-naphthyl)acetonitrile following the protocol of functional group transformations. Subsequent Suzuki coupling of this ester/nitrile derivative with furan-2-boronic acid produced [2-(2-furyl)-1-naphthyl]acetic ester/nitrile which on hydrolysis furnished the corresponding acid derivative. Cyclization of the acid followed by oxidation of the phenol, with Fremy’s salt, produced the tetra-cyclic furoquinone, phenanthro[1,2-*b*]furan-10,11-dione. This method has also been extended for the synthesis of the tricyclic furoquinone, naphtho[1,2-*b*]furan-4,5-dione.

## Introduction

**Figure 1:**
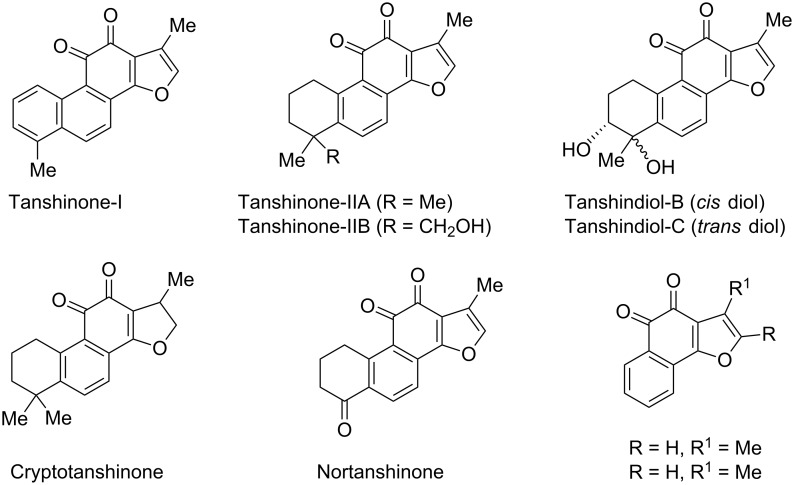
Some biologically active tetra cyclic furoquinone derivatives (isolated from *Dan Shen*) and synthetic tricyclic furoquinones.

Chemistry of furoquinones [1–8], especially the tetra cyclic furoquinones isolated from Chinese red rooted sage *Salvia miltiorrhiza* Bunge known as “*Dan Shen*” in Chinese traditional medicine, attracted the attention of synthetic chemists as well as medicinal chemists due to the various biological activities displayed by such class of compounds. *Dan Shen* is used clinically for treatment of viral hepatitis, cardiac and vascular disorder, hypertension, miscarriage and menstrual disorder [3,9] etc. It has been reported to show potential anticancer activity [10] (against human breast cancer), cytotoxic and antiplatelet aggregation activities [1,11]. It also acts as an antibacterial [1], antioxidant and anti-inflammatory agent [12–14] as well as induces apoptosis in human leukemia cell lines [15–16]. It is believed that broad spectrum of activities of *Dan Shen* are mainly associated with the presence of tetra cyclic furoquinone diterpenoids like Tanshinone I [17–18], Tanshinone IIA and IIB [19–26], Cryptotanshinone [20,22], Nortanshinone [23,27], Tanshindiol [23,27] etc. (Figure 1). Even the tricyclic furoquinones (Figure 1) have also been reported to possess cancer chemo preventive activity [28].

In all of these compounds the key structural unit present is a furophenanthraquinone or a furonaphthoquinone as well as their di/tetra/hexahydro analog.

We have not come across any synthesis of phenanthro[1,2-*b*]furo-10,11-dione (**13**) (the nuclear core structure of Tanshinone-I) though a number of syntheses of naturally occurring tetra cyclic furoquinone diterpenoids have been reported in literature [18–27] in the last few decades. In comparison only very few syntheses of tricyclic furoquinone **18** [29–30] and its derivatives [31–33] have been reported. Encouraged by the broad spectrum biological activity of furoquinones, we aimed to synthesize novel polynuclear furoquinones. Herein we report the synthesis of tetra cyclic furoquinones, phenanthro[1,2-*b*]furan-10,11-dione (**13**) and the tricyclic furoquinone, naphtho[1,2-*b*]furan-4,5-dione (**18**), simulating respectively the A-B-C-D/B-C-D ring of Tanshinone-I, through a novel pathway.

## Results and Discussion

**Figure 2:**
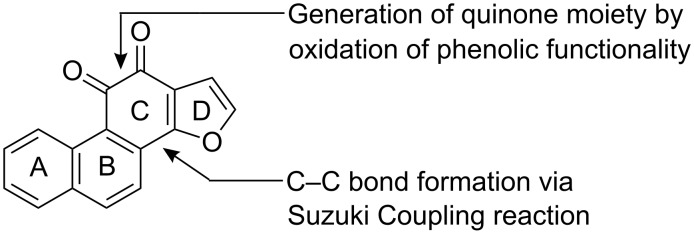
Key strategies for development of C-ring with quinone functionality.

**Scheme 1:**
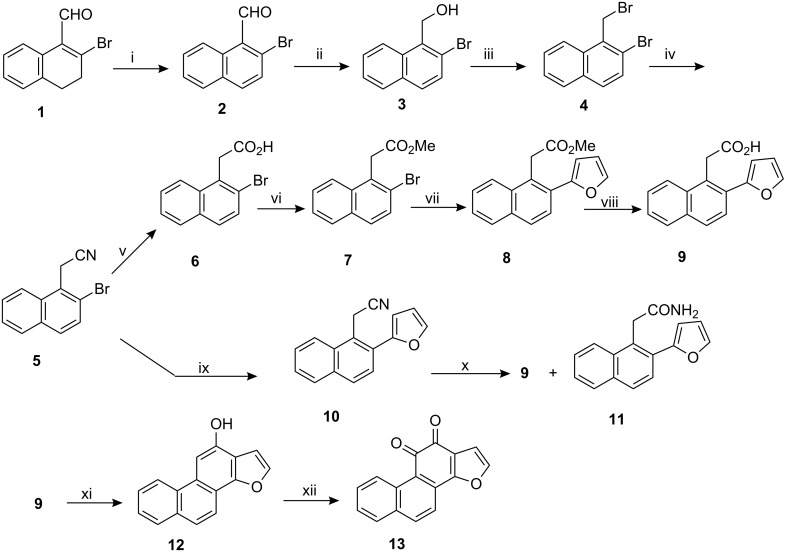
Reagents and conditions: i) DDQ (1.5 equiv), dry benzene, reflux, argon atmosphere, 37 h, 83%. ii) NaBH_4_, EtOH, room temperature, 2 h, 94%. iii) PBr_3_, CCl_4_, 60 °C, 1 h, 82%. iv) KCN, DMF, room temperature, overnight then 1 h at 50 °C, 68%. v) KOH, EtOH, H_2_O, reflux, 23 h, 74%. vi) CH_2_N_2_, ether, 0 °C- room temperature, 95%. vii) furan-2-boronic acid (1.2 equiv.), Et_3_N, DMF, Pd(PPh_3_)_4_ (2 mol %), 110 °C, argon atmosphere, 53 h, 77%. viii) KOH (2 equiv), EtOH, H_2_O, reflux, 15 h, 85%. ix) furan-2-boronic acid (1.2 equiv), Et_3_N, DMF, Pd(PPh_3_)_4_ (2 mol %), 110 °C, argon atmosphere, 37 h, 66%. x) KOH, EtOH, H_2_O, reflux, 45 h, 48%. xi) TFAA, TFA, room temperature, overnight, 71%, xii) Fremy’s salt (3 equiv), MeOH, 1/6 M Na_2_HPO_4_ solution. 0–5 °C, overnight, 48%. (All yields are for purified products only.)

**Scheme 2:**
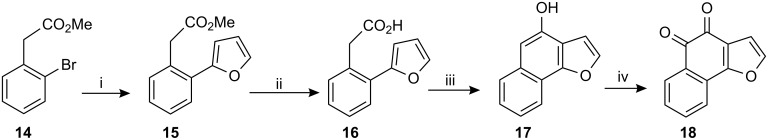
Reagents and conditions: i) furan-2-boronic acid (1.2 equiv), Et_3_N, DMF, Pd(PPh_3_)_4_ (2 mol %), 110 °C, N_2_ atmosphere, 7 h, 84%, ii) KOH, EtOH, H_2_O, reflux, 9 h, 90% iii) TFAA, TFA, room temperature, overnight, 54%, iv) Fremy’s salt (~2.5 equiv), MeOH, 1/6 M Na_2_HPO_4_ solution, 0–5 °C, overnight, 56%. (All yields are for purified products only.)

Our aim is to develop a general route for the synthesis of tricyclic/tetracyclic furoquinones with structural diversity. Our approach is shown in Scheme 1 and Scheme 2. In the synthesis of the tetra cyclic furoquinone **13**, an easily available starting material 2-bromo-3,4-dihydro-1-naphthaldehyde **1** [34] was utilized as A, B ring precursor and commercially available furan 2-boronic acid as D-ring precursor. In the event of the synthesis, the C ring was constructed to reach the target molecule in 8-10 steps. The key steps in our synthesis deal with the formation of aryl-furyl C–C bond via Suzuki reaction [35] and the generation of the quinone functionality by oxidation of a phenolic intermediate (Figure 2).

**Figure 3:**
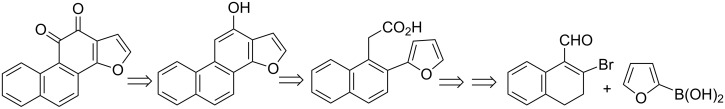
Retro synthesis of tetra cyclic furoquinone **13**.

Retrosynthesis of the molecule showed that the required phenolic compound can easily be achieved in 7–8 steps starting from 2-bromo-3,4-dihydro-1-naphthaldehyde (a substrate easily available by Vilsmeier–Haack reaction on 2-tetralone) and furan-2-boronic acid (a commercially available material) (Figure 3).

When 2-bromo-3,4-dihydro-1-naphthaldehyde (easily obtained in 68% yield by the reaction of 2-tetralone and PBr_3_/DMF in CHCl_3_ at room temperature) was aromatized with DDQ in refluxing benzene, 2-bromo-1-naphthaldehyde (**2**) was produced in excellent yield. Reduction (NaBH_4_/EtOH) of the aldehyde **2** produced the alcohol **3** as a colorless solid, in 94% yield, which on reaction with PBr_3_/CCl_4_ produced the bromide **4** as a light yellow solid. The bromide was then converted (KCN/DMF) to the nitrile derivative **5** which on hydrolysis (KOH/EtOH-H_2_O, reflux) followed by esterification with CH_2_N_2_ furnished methyl 2-(2-bromo-1-naphthyl)acetate **7** in overall good yield. The bromo ester was then subjected to Suzuki reaction with furan-2-boronic acid.

Reaction of compound **7** with furan-2-boronic acid in the presence of Et_3_N and Pd(PPh_3_)_4_ (cat.) in DMF under argon atmosphere produced methyl [2-(2-furyl)-1-naphthyl]acetate **8** (77%) which when hydrolyzed furnished [2-(2-furyl)-1-naphthyl]acetic acid (**9**) in 85% yield. The acid **9** was also synthesized via hydrolysis of the nitrile derivative **10** which in turn was obtained in 66% yield, by direct Suzuki reaction of **5** with furan-2-boronic acid. However in this case as the intermediate amide **11** produced was resistant to further hydrolysis, the reaction required prolonged reflux and also the yield was relatively poor. Even after reflux of the nitrile derivative **10** in KOH/EtOH-H_2_O for 45 h 20% of the amide **11** was recovered along with 48% of the desired carboxylic acid. Change of solvent and conditions (e.g., replacement of ethanol with other high boiling alcohols or use of THF as co-solvent and higher temperature) produced no better result. The next step was the introduction of the phenol functionality. In this case our early attempt to prepare the phenol **12** by PPA cyclization of the acid **9** was unsuccessful. We however successfully prepared the phenol as a light yellow solid by cyclization of the acid **9** with trifluoroacetic acid (TFA) and trifluoroacetic anhydride (TFAA) at room temperature in 71% yield. The phenol **12** was finally oxidized (with Fremy’s salt) [36–38] to the *o*-quinone **13** to complete the synthesis of phenanthro[1,2-*b*]furan-10,11-dione. The compounds were characterized by usual spectroscopic and analytical methods (^1^H NMR, ^13^C NMR, IR, HRMS etc.).

**Figure 4:**
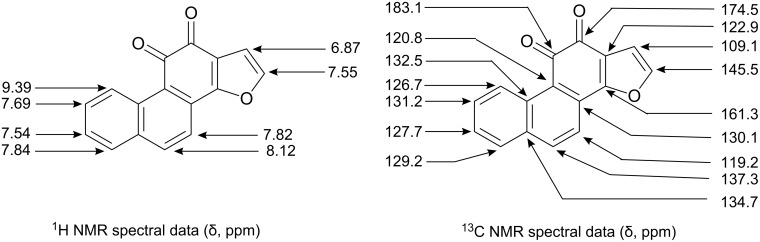
Assignment of chemical shifts (^1^H NMR and ^13^C NMR) of compound **13**.

The developed pathway was then applied for the synthesis of tricyclic furoquinone **18** (naphtho[1,2-*b*]furan-4,5-dione) starting from methyl (2-bromophenyl)acetate **14** and furan-2-boronic acid (Scheme 2).

When methyl (2-bromophenyl)acetate **14** was subjected to Suzuki reaction with furan-2-boronic acid, produced [2-(2-furyl)phenyl]acetic ester **15** as a pale yellow liquid in 84% yield. Subsequent alkaline hydrolysis of **15** produced the carboxylic acid **16** as a white solid in 90% yield. Synthesis of the furonaphthol **17** was achieved in good yield, by cyclization of the carboxylic acid **16** with TFA and TFAA at room temperature. Finally oxidation of the naphthol derivative **17**, with Fremy’s Salt, resulted in the formation of naphtho[1,2-*b*]furan-4,5-dione (**18**), as a red solid, in 56% yield. The compounds have been characterized by usual spectroscopic analysis (NMR, IR and HRMS data) as well as by analogy with literature report [30].

In general we have developed a novel pathway for the synthesis of polynuclear furoquinones simulating BCD/ABCD ring of natural furoquinone (Tanshinone-I) and we believe the method has great potential towards the synthesis of various polynuclear furoquinone derivatives bearing electron donating/electron withdrawing functionality within its framework as bromoaldehyde derivatives bearing such groups can easily be obtained from corresponding ketones by Vilsmeier–Haack reaction. Very recently we have reported a general method for the synthesis of β-(2-furyl)-α,β-unsaturated aldehydes [39] via Suzuki reaction of β-bromo-α,β-unsaturated aldehydes. These substrates can be used for the synthesis of various non-natural tricyclic and “U-shaped” tetra cyclic furoquinone derivatives. The work is under progress and the results will be published in due course.

## Experimental

Furan-2-boronic acid, 2-tetralone and tetrakis(triphenylphosphine)palladium(0) and DDQ were purchased from Sigma-Aldrich (U.S.A). Trifluoroacetic anhydride was purchased from Alfa Aesar (Lancaster). 2-Bromo-3,4-dihydronaphthalene-1-carbaldehyde was prepared from 2-tetralone by Vilsmeier-Haack reaction. Solvents were dried following standard literature procedure. Fremy’s salt (potassium nitrosodisulfonate) was prepared in the laboratory as per literature procedure [36]. ^1^H NMR spectra were recorded on Bruker 500 MHz (at Chemgen Pharma, Kolkata) and Bruker 400 MHz (at Chembiotek International, Kolkata) and Bruker 300 MHz (IACS, Kolkata) NMR spectrometer respectively. ESI mass spectra were recorded on a micro mass Q-TOF mass spectrometer (serial no. YA 263) at IACS, Kolkata. IR spectral data were obtained from a JASCO FT/IR680 PLUS Spectrometer.

### [2-(Furan-2-yl)-naphthalen-1-yl]acetic acid methyl ester (**8**)

A mixture of compound **7** (150 mg, 0.54 mmol), furan-2-boronic acid (75 mg, 0.64 mmol) and Et_3_N (0.5 mL, 3.6 mmol) was degasified with argon for 25 minutes. Now to it the catalyst Pd(PPh_3_)_4_ (~15 mg, 2 mol %) was added. The mixture was then heated with stirring under argon at 110 °C till the reaction was completed (~53 h). After cooling to room temperature, the mixture was poured into cold water and extracted with ether. Organic layer was washed successively with NaHCO_3_ solution, 5% brine and dried (Na_2_SO_4_) and solvent removed. Crude product thus obtained was then purified by column chromatography [silica gel/ pet. ether (60–80 °C) and ethyl acetate mixture, 10:1]. Compound **8** was obtained as a white solid (110 mg) in 77% yield, m.p., 109–111 °C. IR(KBr) ν_max_ : 1737.6 cm^−1^; ^1^H NMR (400 MHz, CDCl_3_) δ: 3.72 (3H, s), 4.33 (2H, s), 6.53 (1H, dd, *J* = 1.7 and 3 Hz), 6.67 (1H, d, *J* = 3 Hz), 7.50 (1H, t, *J* = 7.2 Hz), 7.56 (1H, d, *J* = 7.2 Hz), 7.57 (1H, br s), 7.72 (1H, d, *J* = 8.6 Hz), 7.82 (1H, d, *J* = 9.4 Hz), 7.84 (1H, d, *J* = 9.3 Hz), 8.01 (1H, d, *J* = 8.4 Hz), ppm.

### [2-(Furan-2-yl)-naphthalen-1-yl]acetic acid (**9**)

A mixture of compound **8** (100 mg, 0.38 mmol), KOH (43 mg, 0.76 mmol), 2 mL water and 2 mL ethanol was refluxed on a water bath for 15 h. Excess EtOH was distilled out as much as possible and then the residue was diluted with 2–3 mL of water. Neutral part was extracted with ether. Aqueous alkaline part was cooled in ice and acidified with HCl. Separated solid was thoroughly extracted with ethyl acetate. After usual work up, 80 mg (85%) of the acid **9** was obtained as white solid, m.p., 197–199 °C. IR (KBr) ν_max_ 1692.2 cm^−1^. (Treatment of the acid **9** with diazomethane in ether produced the methyl ester derivative identical with compound **8**.)

### Phenanthro[1,2-*b*]furan-11-ol (**12**)

A mixture of compound **9** (100 mg, 0.39 mmol), 3 mL trifluoroacetic anhydride, 0.9 mL trifluoroacetic acid was stirred at room temperature overnight protecting from moisture. The mixture was then poured in ice cold saturated NaHCO_3_ solution and stirred well and extracted thoroughly with CH_2_Cl_2_. The organic layer was washed with aq. NaHCO_3_ solution and finally with H_2_O and dried (Na_2_SO_4_). Removal of solvent afforded the crude product which was purified by column chromatography [silica gel/pet. ether (60–80 °C) and ethyl acetate mixture, 19:1] to furnish 65 mg (71%) of **12** as a light yellow solid, m.p., 175–177 °C. IR (KBr) ν_max_ 3235, 3302 cm^−1^; ^1^H NMR (500 MHz, CDCl_3_) δ: 5.47 (1H, s), 7.04 (1H, br s), 7.58 (1H, t, *J* = 7.4 Hz), 7.63 (1H, t, *J* = 7.5 Hz), 7.75 (1H, d, *J* = 8.8 Hz), 7.78 (1H, br s), 7.85 (1H, s), 7.91 (1H, d, *J* = 7.8 Hz), 8.22 (1H, d, *J* = 8.8 Hz), 8.55 (1H, d, *J* = 8.2 Hz) ppm.

### Phenanthro[1,2-b]furan-10,11-dione (**13**)

To a stirred solution of potassium nitrosodisulfonate (Fremy’s salt) (170 mg, 0.65 mmol) in 12 mL 1/6 (M) Na_2_HPO_4_ solution taken in a 50 mL round bottomed flask, a solution of the furonaphthol derivative **12** (50 mg, 0.21 mmol) in 6 mL methanol was added drop wise. Stirring was continued at 0–5 °C for 2 h and then left overnight in freeze. The dark red solid separated was filtered and purified by column chromatography [silica gel/ pet. ether (60–80 °C) and ethyl acetate, 10:1]. An analytical sample was prepared further by recrystallisation from pet ether–ethyl acetate mixture. Yield, 25 mg (48%), m.p., 178–180 °C. IR (KBr) ν_max_ 1686, 1667 cm^−1^; ^1^H NMR (500 MHz, CDCl_3_) δ: 6.87 (1H, br s), 7.53 (1H, d, *J* = 7.5 Hz), 7.55 (1H, br s), 7.70 (1H, br t, *J* = 7.5 Hz), 7.81–7.85 (2H, a doublet and a triplet merged together), 8.12 (1H, d, *J* = 8.5 Hz), 9.40 (1H, d, *J* = 8.5 Hz) ppm; ^13^C NMR (125 MHz, CDCl_3_) δ: 109.11, 119.16, 120.79, 122.88, 126.66, 127.66, 129.20, 130.07, 131.20, 132.47, 134.65, 137.33, 145.54, 161.31, 174.53, 183.07 ppm; HRMS (ESI, 70 eV): *m/z* = 271.0372 [M^+^+Na] (calculated mass for C_16_H_8_O_3_Na: 271.0371 [M^+^+Na]).

### Naphtho[1,2-*b*]furan-4,5-dione (**18**)

To a stirred solution of potassium nitrosodisulfonate (Fremy’s salt) (170 mg, 0.63 mmol) in 5 mL 1/6 (M) Na_2_HPO_4_ solution taken in a 25 mL round bottomed flask, a solution of the furonaphthol **17** (50 mg, 0.27 mmol) in 2 mL methanol was added drop wise. Stirring was continued at 0–5 °C for 2 h and then left overnight in freeze. The brick red solid separated was filtered to obtain 35 mg of crude product which on purification by column chromatography (silica gel/pet. ether–ethyl acetate, 5:1) afforded 30 mg (56%) of the title compound (**18**). An analytical sample was prepared by further recrystallisation from pet. ether–EtOAc mixture; m.p., 205–206 °C (lit. [30] m.p 213–215 °C ). IR(KBr) ν_max_ 1674.9 cm^−1^ (br, strong); ^1^H NMR (500 MHz, CDCl_3_) δ: 6.88 (1H, d, *J* = 1.8 Hz), 7.48 (1H, t, *J* = 7.5 Hz), 7.51 (1H, d, *J* = 1.8 Hz), 7.67 (1H, t, *J* = 7.5 Hz), 7.74 (1H, d, *J* = 7.5 Hz), 8.1 (1H, d, *J* = 7.7 Hz) ppm; ^13^C NMR (125 MHz, CDCl_3_) δ: 108.94, 121.56, 122.41, 128.49, 128.81, 130.36, 130.64, 135.48, 145.13, 160.64, 174.54, 180.56 ppm. HRMS (ESI, 70 eV): *m/z* = 199.0399 [M^+^+H] (calculated mass for C_12_H_7_O_3_: 199.0391 [M^+^+H]).[lit. [30]: ^1^H NMR (300 MHz, CDCl_3_) δ: 8.09 (1H, d, *J* = 7.9 Hz), 7.73–7.62 (2H, m), 7.49 (1H, d, *J* = 2.0 Hz), 7.45 (1H, m), 6.86 (1H, d, *J* = 2.0 Hz)].

## Supporting Information

Supporting information features experimental procedures, analytical data and NMR spectra for some selected compounds.

File 1 Experimental procedures

File 2 ^1^H and ^13^C NMR Spectra.
