# Supplementary material for: Studies on polynuclear furoquinones. Part 1: Synthesis of tri- and tetra-cyclic furoquinones simulating BCD/ABCD ring system of furoquinone diterpenoids
Source: Beilstein J Org Chem. 2009 Sep 29;5:47. doi: 10.3762/bjoc.5.47 (PMC2779660; doi:10.3762/bjoc.5.47)
Supplement: File 1 — Experimental procedures [file Beilstein_J_Org_Chem-05-47-s001.doc]

Supporting Information: Experimental procedures for some selected compounds

Studies on polynuclear furoquinones. Part 1: Synthesis of tri- and tetra-cyclic furoquinones simulating BCD/ABCD ring system of furoquinone diterpenoids.

Faruk Hasan Shaik, Gandhi Kumar Kar*

Address: Department of Chemistry, Presidency College, 86/1 College Street, Kolkata-700073, India

*Corresponding author

**2-Bromonaphthalene-1-carbaldehyde (2)**:

A mixture of 1.0 g (4.22 mmol) of compound **1**, 1.9 g (6.33 mmol) of DDQ in 20 mL dry benzene was taken in a 100 mL round bottomed flask, and refluxed on a water bath under argon atmosphere for 37 h. Crude reaction mixture was filtered through 60-120 mesh silica gel column and the filtrate was then washed thoroughly with saturated Na_2_CO_3_ solution and finally with water. The organic layer was collected, dried (anhydrous Na_2_SO_4_) and solvent removed. The solid obtained was recrystallised from pet. ether to yield compound **2** (830 mg, 83%), m.p., 92–93 °C as a light yellow solid. IR (KBr) ν_max_ : 1681.6 cm^-1^. ^1^H NMR (400 MHz, CDCl_3_) δ: 7.58 (1H, brt, J = 7.2), 7.65–7.68 (1H, m), 7.69 (1H, d, J = 8.8 Hz), 7.84 (1H, brd, J = 8.4 Hz), 7.87 (1H, d, J = 8.8 Hz), 9.09 (1H, d, J = 8.8 Hz), 10.75 (1H, s) ppm.

**(2-Bromonaphthalen-1-yl)methanol (3)**:

To a cold (5–10 °C) stirred solution of the 2-bromo-1-naphthaldehyde (**2**) (300 mg, 1.19 mmol) in 15 mL ethanol taken in 50 mL round bottomed flask, 35 mg (1 mmol) of NaBH_4_ was added pinch by pinch. Stirring was continued at room temperature for further 2 hrs. Excess ethanol was removed by distillation and the residue was diluted with 3 mL ice water containing 3–4 drops of dil. HCl. The solid separated was filtered and thoroughly washed with ice cold water. It was further purified by column chromatography (silica gel/ pet. ether (60–80 °C) & ethyl acetate mixture, 10:1) to obtain 282 mg (94%) of compound **3** as a white solid; m.p., 127–129 °C. IR (KBr) ν_max_ : 3100–3345 (br) cm^-1^; ^1^H NMR (400 MHz, CDCl_3_) δ: 1.19 (1H, t, J = 6.1 Hz), 5.30 (2H, d, J = 6.1 Hz), 7.52 (1H, brt, J = 7.2 Hz), 7.59 (1H, brt, J = 7.2 Hz), 7.61 (1H, d, J = 8.8 Hz), 7.67 (1H, d, J = 8.8 Hz), 7.83 (1H, d, J = 8 Hz), 8.24 (1H, d, J = 8.4 Hz) ppm.

**2-Bromo-1-bromomethylnaphthalene (4)**:

A mixture of compound **3** (300 mg, 1.27 mmol), PBr_3_ (160 mg, 0.59 mmol) and 5 mL CCl_4_ was stirred at 60 °C for 1 hr protecting from moisture. It was then cooled to room temperature, poured into crushed ice and extracted with CHCl_3_ (2x 10 mL). The CHCl_3_ layer was washed with saturated NaHCO_3_ solution followed by water. Organic layer was separated, dried over anhyd.Na_2_SO_4_ and solvent removed to furnish the bromide **4** as a faint yellow solid which was purified by column chromatography [silica gel/10:1 pet. ether (60–80 °C)-ethyl acetate]. Yield, 252 mg (82%); m.p., 97–98 °C. ^1^H NMR (400MHz, CDCl_3_) δ: 5.13 (2H, s), 7.54 (1H, brt, J = 7.2 Hz), 7.61 (1H, d, J = 8.8 Hz), 7.66 (1H. brt, J = 7.2 Hz), 7.68 (1H, d, J = 8.8 Hz), 7.84 (1H, d, J = 8.0 Hz), 8.11 (1H, d, J = 8.4 Hz) ppm; ^13^C NMR (75 MHz, CDCl_3_) δ: 30.27, 123.74, 126.47, 127.71, 128.85, 130.26, 130.55, 132.07, 132.15, 132.80 ppm.(peak due to one quaternary carbon was not observed in this NMR. Possibly it is merged with some other signal); HRMS (ESI, 70 eV): *m/z* = 300.9041 [M^+^+H] (calculated mass for C_11_H_8_Br_2_: 300.9045 [M^+^+H]).

**(2-Bromo-naphthalen-1-yl)acetonitrile (5):**

In a 50 mL round bottomed flask fitted with anhydrous CaCl_2_ guard tube, 270 mg (0.9 mmol) of bromide **4** and 2 mL dry DMF was taken. To this 120 mg (1.85 mmol) of potassium cyanide was added and stirred at room temperature. for overnight and then 1 h at 50°C. The reaction mixture was then poured into 5 mL ice water and extracted with ether. Ether part was washed thoroughly with ice-cold brine solution and finally once with ice cold H_2_O. Organic part was collected; dried (anhydrous Na_2_SO_4_) and removal of solvent afforded the crude cyanide derivative **5**. It was purified by column chromatography [silica gel/ pet. ether (60–80 °C) and EtOAc, 20:1]. Yield, 150 mg (68%); white solid; m.p., 127–129 °C. IR (KBr) ν_max_: 2245.7 cm^-1^; ^1^H NMR (400 MHz, CDCl_3_) δ: 4.34 (2H, s), 7.57 (1H, m), 7.64–7.67 (2H, m), 7.72 (1H, d, J = 8.8 Hz), 7.87 (1H, d, J = 7.9 Hz), 7.8 (1H, d, J = 8.7 Hz) ppm.

**(2-Bromo-naphthalen-1-yl)acetic acid (6)**:

In a 25 mL round bottomed flask attached with a reflux condenser, 200 mg (0.81 mmol) of compound **5**, 3 mL 20% KOH and 10 mL methanol was taken and refluxed at 100 °C for 23 h. Excess methanol was removed by distillation and the residue was diluted with ice-water and neutral part was extracted with ether. Aqueous part was acidified with 1:1 HCl under ice cold condition. Solid appeared was filtered through suction. The residue was again dissolved in NaHCO_3_ solution and filtered. Filtrate was acidified with conc. HCl in ice cold condition and solid separated was filtered. It was recrystallised from pet. ether and ethyl acetate mixture. Yield, 160 mg (74%); white solid, m.p., 187–188 °C. IR (KBr) ν_max_: 1693.3 cm^-1^.

**(2-Bromo-naphthalen-1-yl)acetic acid methyl ester (7)**:

160 mg (0.6 mmol) of compound **6** was added pinch by pinch, to an ether solution of diazomethane (generated from 200 mg nitrosomethylurea at 0 °C. Removal of solvent afforded the ester **7** which was recrystallised from pet. ether (60–80 °C) to produce pure **7** as colorless solid. Yield 160 mg (95%); m.p., 96–97 °C. IR (KBr) ν_max_: 1732.7 cm^-1^ ;

^1^H NMR (500 MHz, CDCl_3_) δ: 3.70 (3H, s), 4.36 (2H, s), 7.51 (1H, t, J = 7.3 Hz), 7.56 (1H, t, J = 7.2 Hz), 7.66 (2H, brs), 7.83 (1H, d, J = 7.8 Hz), 7.94 (1H, d, J = 8.4 Hz) ppm.

**[(2-Furan)-2-yl-naphthalen-1-yl]acetonitrile (10):**

A mixture of compound **5** (150 mg, 0.61 mmol), furan-2-boronic acid (82 mg, 0.73 mmol), Et_3_N (0.5 mL, 3.6 mmol) and DMF (1.5 mL) was degassed by argon for 30 min and then to it the catalyst, Pd(PPh_3_)_4_ (15mg, ~2 mol %) was added. The mixture was then heated with stirring at 110 °C under argon atmosphere for 37 hrs. After cooling to room temperature it was poured in cold water and extracted with ether. After usual work up followed by purification with column chromatography (silica gel/ pet. ether–ethyl acetate, 10:1) afforded 95 mg (66%) of **10** as a light yellow solid (m.p.,103–104 °C). IR(KBr) ν_max_ 2247.6 cm^-1^ ; ^1^H NMR (500MHz, CDCl_3_) δ: 4.30 (2H, s), 6.60 (1H, d, J = 1.5 Hz) , 6.76 (1H, d, *J =* 2.8 Hz), 7.56 (1H, t, J = 7.4 Hz), 7.66 (2H, m), 7.70 (1H, d, J = 8.6 Hz), 7.88 (2H, m), 8.07 (1H, d, *J =* 8.5 Hz) ppm; ^13^C NMR (125 MHz, CDCl_3_) δ: 18.63, 110.48, 111.79, 118.14, 122.37, 123.41, 125.96, 126.58, 127.77, 128.88, 129.19, 131.87, 133.15, 143.39, 152.57 ppm ( signal due to one carbon was not observed , possibly it is merged with any other signal); HRMS (ESI, 70 eV): *m/z* = 256.0736 [M^+^+Na] (calculated mass for C_16_H_11_ONNa: 256.0739 [M^+^+Na]).

**Hydrolysis of the nitrile 10 to prepare the acid 9:**

90 mg (0.39 mmol) of the nitrile **10** on hydrolysis with KOH ( 20% solution, 1 mL) and 3–4 mL of ethanol under reflux at 100 °C for 45 h afforded, after usual work up, the acid **9** ( 43 mg, 48%, m.p., 196–198 °C ) and the amide **11** ( 20 mg, 20%) as neutral part. Compound **11:** white solid, m.p., 237–238 °C. IR (KBr) ν_max_ 1654.6, 3191.6, 3378.6 cm^-1^; ^1^H NMR (500 MHz, CDCl_3_)δ: 4.25 (2H,s), 5.36 (1H, brs), 5.56 (1H, brs), 6.58 (1H, brs), 6.68 (1H, brs), 7.54 (1H, t, J = 7.5 Hz), 7.60 ( 1H, brt, J = 7.5 Hz), 7.61 (1H, brs), 7.74 (1H, d, J = 8.5 Hz), 7.86 (1H, brd, J = 7.8 Hz), 7.87 (1H, brd, J = 7.8 Hz), 8.13 (1H, brd, J = 8.5 Hz) ppm.

**Methyl [2-(2-furyl)phenyl]acetate (15)**:

A mixture of methyl 2-bromophenylacetate (**14**) (690 mg, 3.01 mmol), furan-2-boronic acid (403 mg, 3.62 mmol), Et_3_N (0.9 mL, 6.5 mmol) and DMF (2 mL) taken in a two necked flask, was degasified by nitrogen for 30 min. Then Pd(PPh_3_)_4_ catalyst (70 mg, ~2 mol%,) was added to the reaction mixture under nitrogen atmosphere. Then reaction mixture was heated with stirring at 110 °C under nitrogen atmosphere for 7 h (completion of the reaction was monitored by TLC). The mixture was then cooled and poured into ice water (~20 mL) with stirring and extracted with ether (3 x 20 mL). Ether part was washed successively with 5% brine, saturated NaHCO_3_ solution and finally thoroughly with water and dried (anhydrous Na_2_SO_4_). Removal of the solvent afforded the crude product which was purified by column chromatography (silica gel/ pet. ether (60–80 °C)–ethyl acetate mixture, 10:1) to obtain compound **15** as a pale yellow oil

(545 mg, 84%). IR (KBr) ν_max_: 1729.8 cm^-1^. ^1^H NMR (400 MHz, CDCl_3_) δ 3.67 (3H, s), 3.87 (2H, s), 6.47 (1H, dd, J = 3 Hz & 1.8 Hz), 6.56 (1H, d, J = 3 Hz), 7.27–7.34 (3H, m), 7.48 (1H, d, J = 1.3 Hz), 7.61 (1H, d, J = 7.4 Hz) ppm.

**[2-(2-furyl)phenyl]acetic acid (16)**:

To a solution of **15** (250 mg, 1.16 mmol) in EtOH (3.5 mL), 1 ml 20% aqueous solution of KOH was added. The mixture was refluxed on a water bath for 9 h. The excess EtOH was then removed and the residue was diluted with 5 mL of ice water and extracted with ether to remove the neutral part if any. The aqueous alkaline part was cooled in ice-salt mixture and acidified with 5N HCl. The white ppt. appeared was filtered under suction, washed thoroughly with ice-water and dried.

Yield, 182 mg (90%); m.p., 128–129 °C. IR(KBr) ν_max_: 1692.2 cm^-1^. HRMS (ESI, 70 eV): *m/z* = 225.0524 [M^+^+Na] (calculated mass for C_12_H_10_O_3_Na: 225.0528 [M^+^+Na]).

**Naphtho[1,2-*b*]furan-4-ol (17)**:

In a 5 ml round bottomed flask, the acid **16** (200 mg, 0.99 mmol) was taken and to it 2.5 mL trifluoroacetic anhydride and 0.5 mL of trifluoroacetic acid was added. The mixture was stirred at room temperature for overnight protecting from moisture. The reaction mixture was then poured into ice cold saturated NaHCO_3_ solution with stirring and extracted with dichloromethane (3x10 mL). DCM layer was washed successively with NaHCO_3_ solution and water and then dried over anhydrous Na_2_SO_4_. Solvent was distilled out and the crude product thus obtained was purified by column chromatography (silica gel/ benzene) to furnish compound **17** as a faint brownish solid. . Yield, 100 mg (54%); m.p., 109–111 °C. IR(KBr) ν_max_ 3267 cm^-1^; ^1^H NMR (400 MHz, CDCl_3_) δ: 5.32 (1H, s), 6.93 (1H, s), 6.99 (1H, d, J = 2 Hz), 7.41–7.46(2H, m), 7.74 (1H, d, J = 2 Hz), 7.73–7.77 (1H, m), 8.19–8.23 (1H, m) ppm; ^13^C NMR (125 MHz, CDCl_3_) δ: 103.85, 104.62, 116.05, 117.78, 120.15, 123.93, 125.81, 126.68, 132.39, 143.78, 147.98, 152.19 ppm; HRMS (ESI, 70 eV): *m/z* = 185.0595 [M^+^+H] (calculated mass for C_12_H_9_O_2_: 185.0597 [M^+^+H]).
