# Supplementary material for: Studies on polynuclear furoquinones. Part 1: Synthesis of tri- and tetra-cyclic furoquinones simulating BCD/ABCD ring system of furoquinone diterpenoids
Source: Beilstein J Org Chem. 2009 Sep 29;5:47. doi: 10.3762/bjoc.5.47 (PMC2779660; doi:10.3762/bjoc.5.47)

**Supporting Information: ^1^H NMR and ^13^C NMR spectra of selected compounds**

**Studies on polynuclear furoquinones. Part 1: Synthesis of tri- and tetra-cyclic furoquinones simulating BCD/ABCD ring system of furoquinone diterpenoids.**

Faruk Hasan Shaik, Gandhi Kumar Kar*

Department of Chemistry, Presidency College, 86/1 College Street, Kolkata-700073, India

*Corresponding author


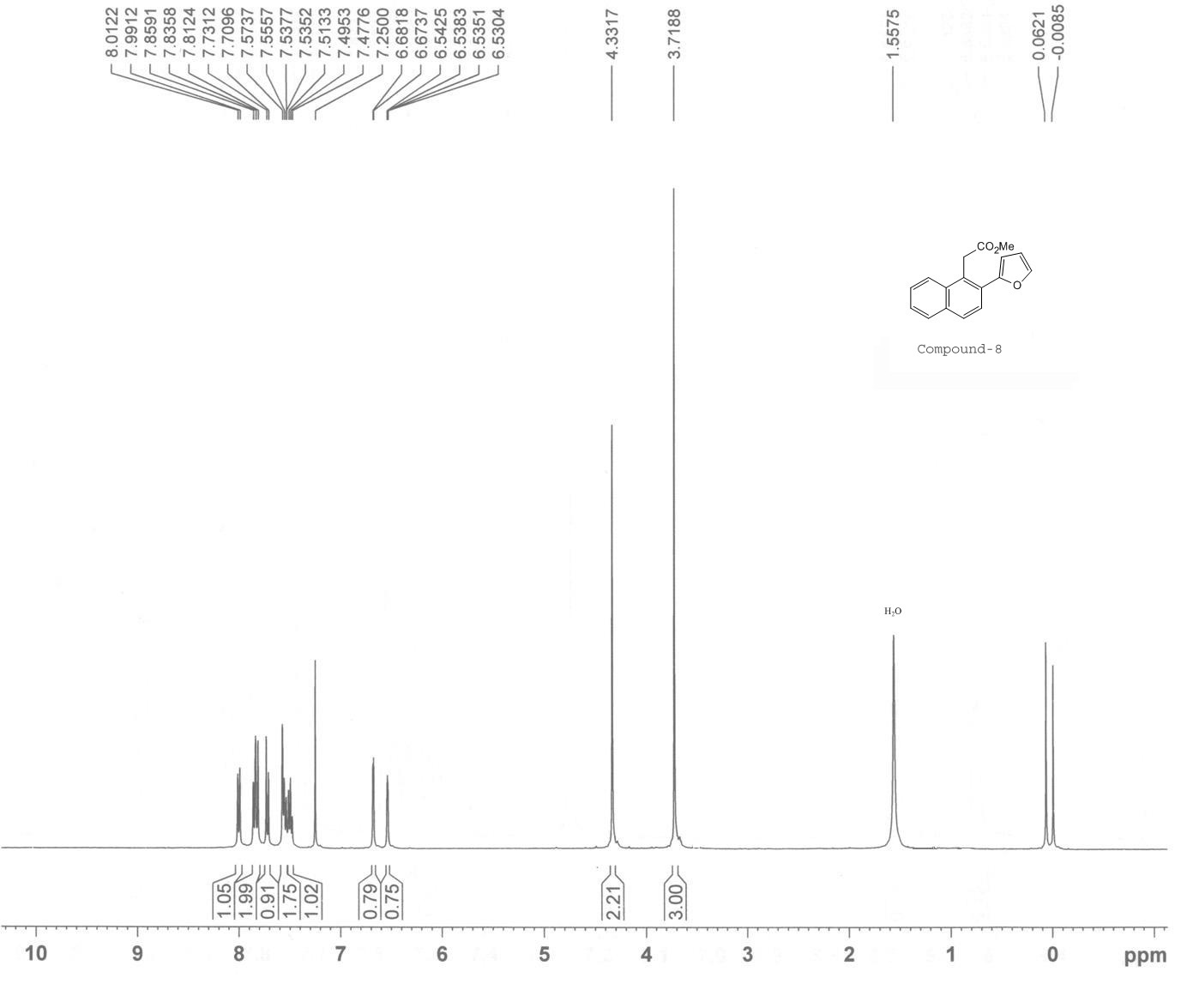


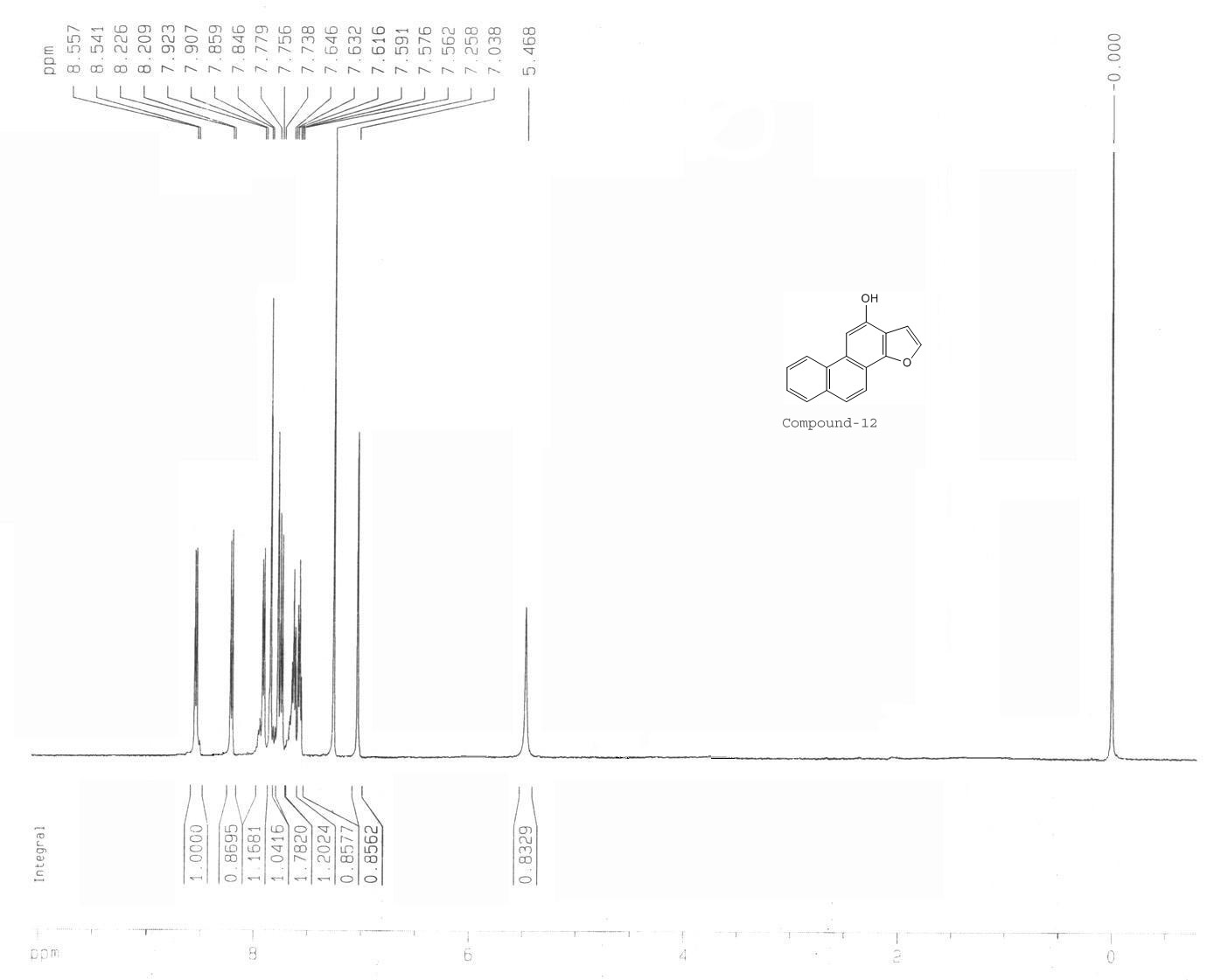


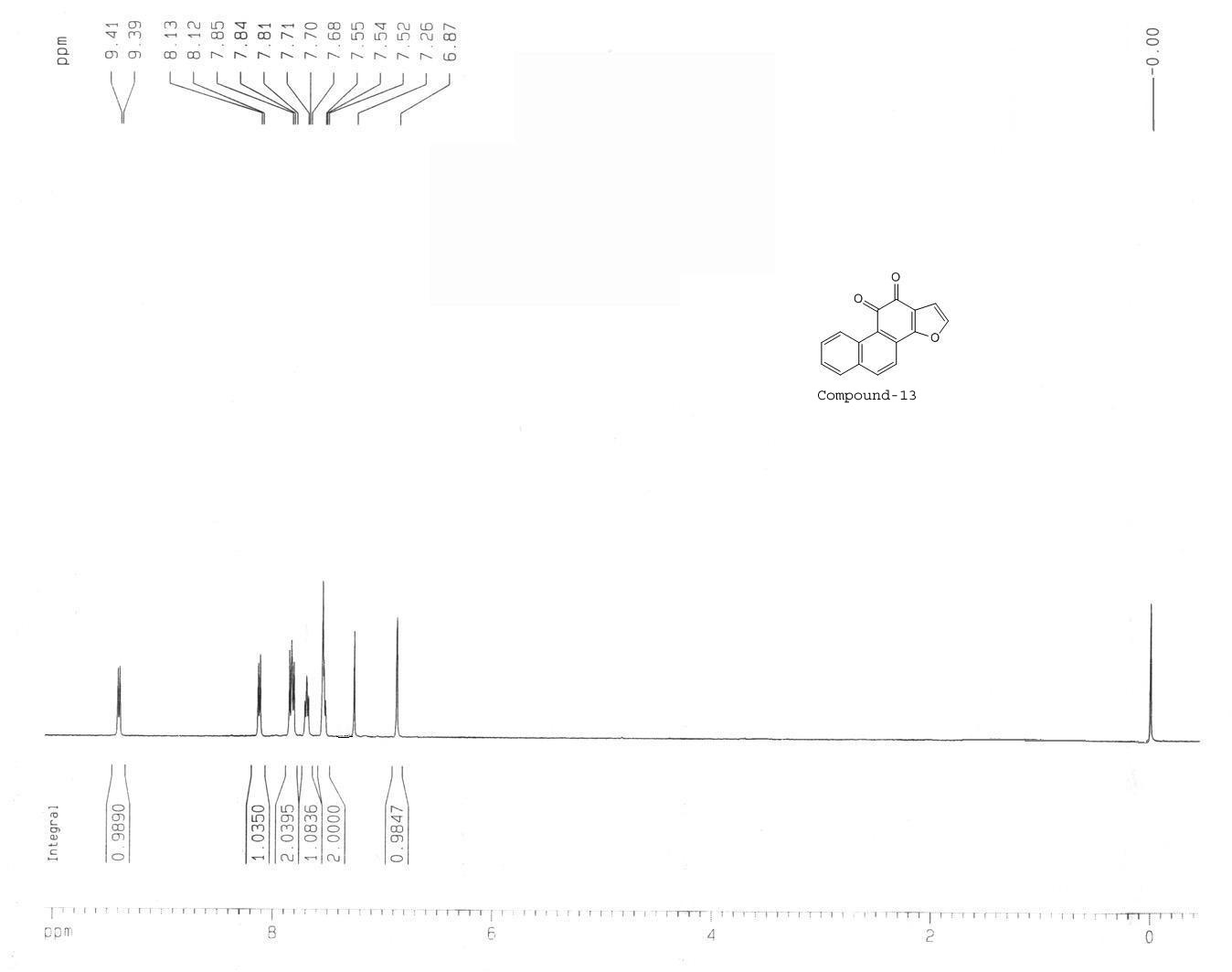


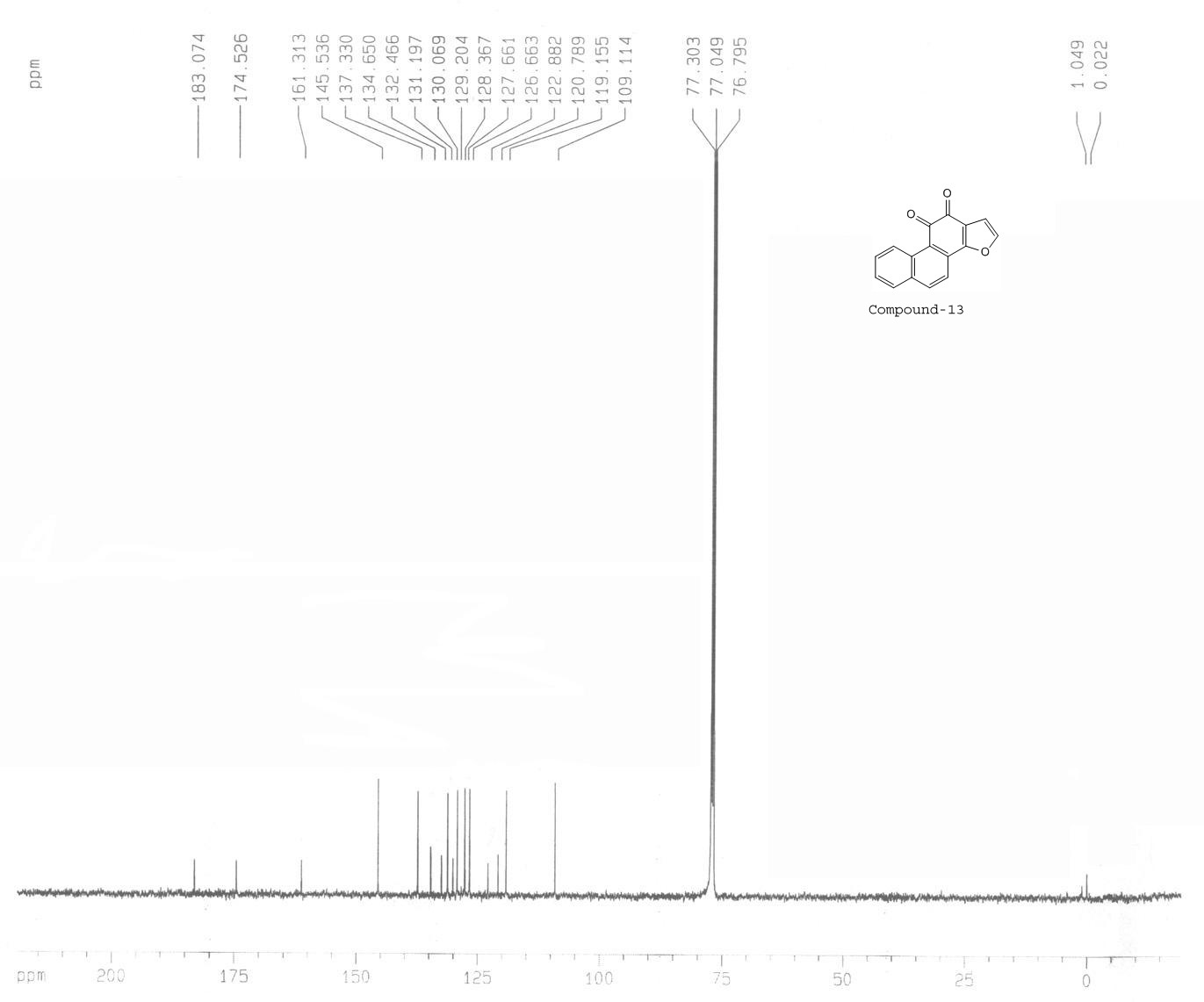


HSQC Spectra of compound-**13**:


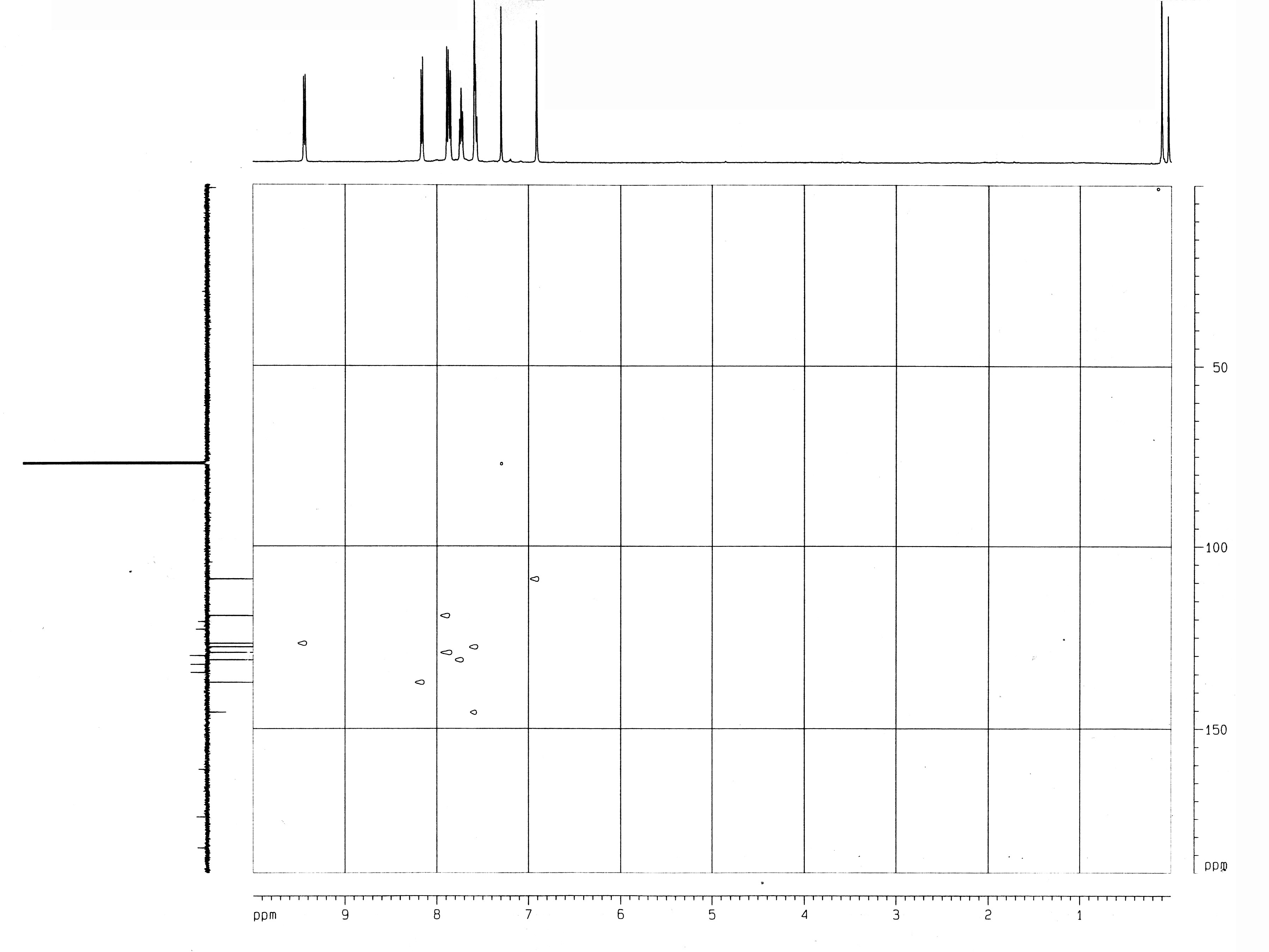


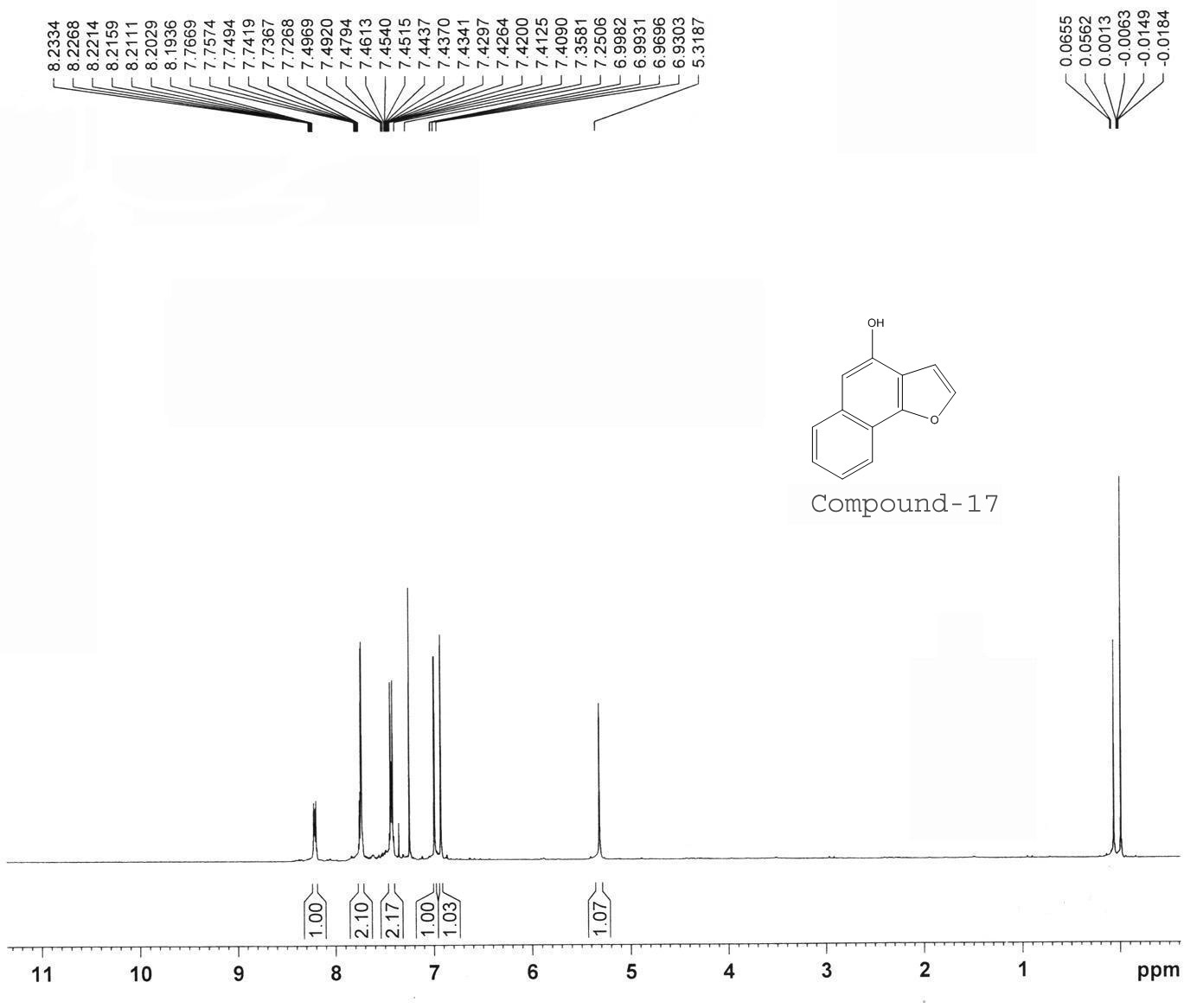


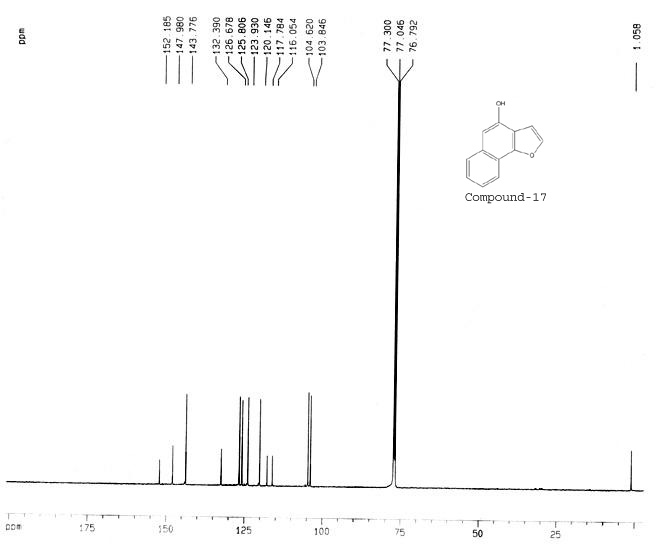


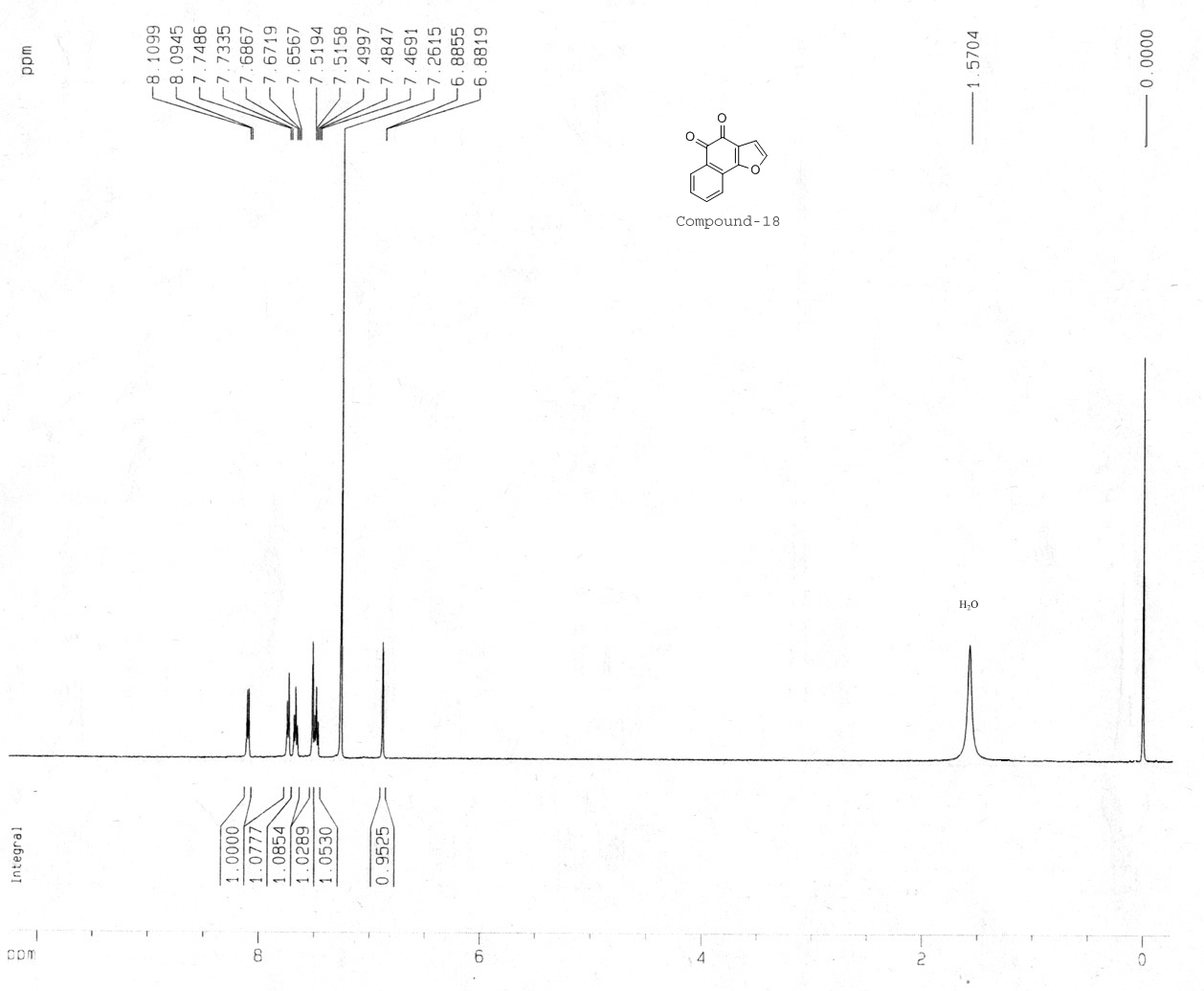


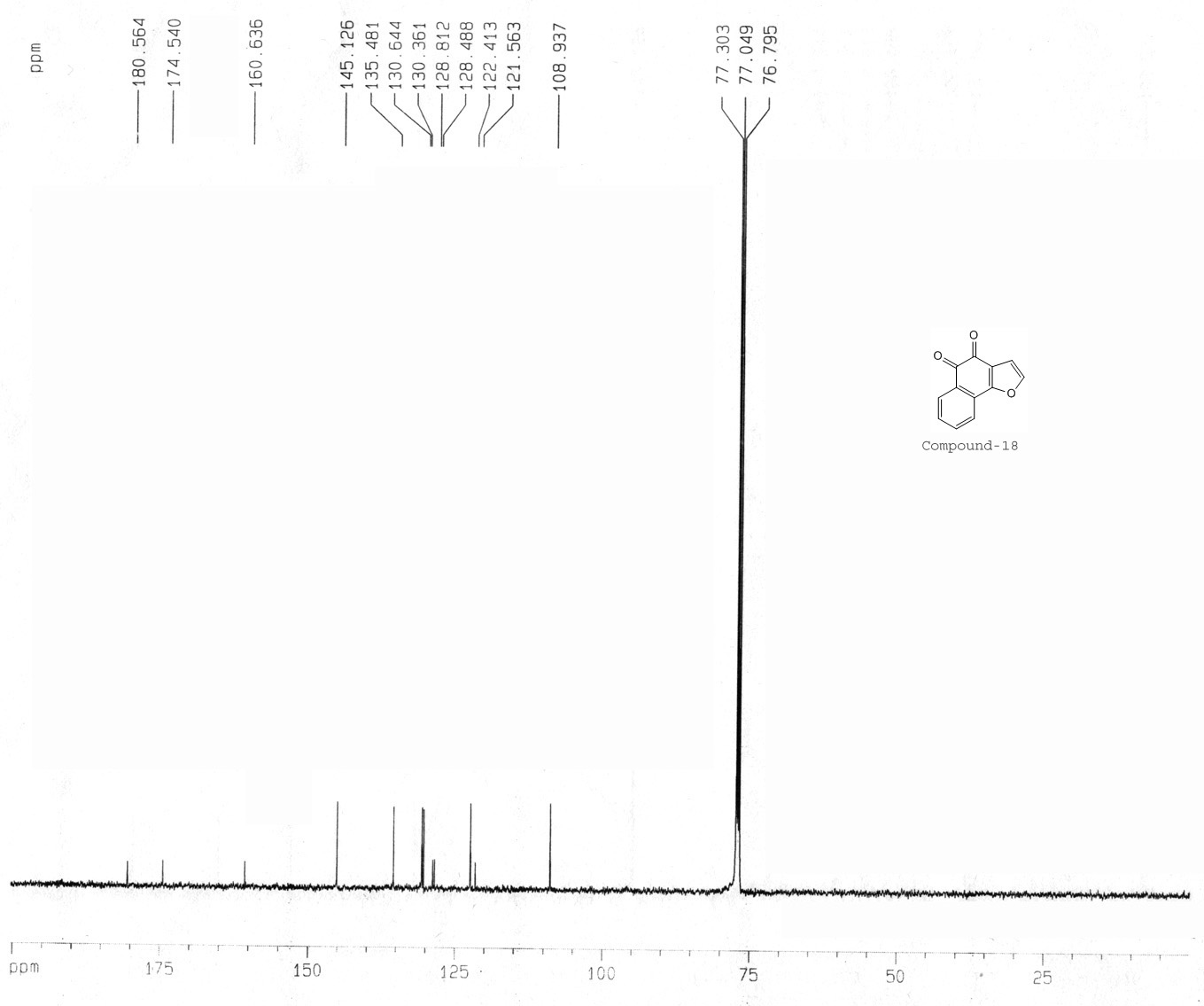

Supplement: File 2 — 1H and 13C NMR Spectra. [file Beilstein_J_Org_Chem-05-47-s002.doc]
